# Community Health Agents Advancing Women’s Empowerment: A Qualitative Data Analysis

**DOI:** 10.1007/s10900-022-01107-2

**Published:** 2022-06-24

**Authors:** Elizabeth M. Allen, Ariel Frisancho, Claudia Llanten, Maren E. Knep, Michael J. Van Skiba

**Affiliations:** 1 Department of Public Health, St. Catherine University, 2004 Randolph Ave, St. Paul, MN 55104 USA; 2 Catholic Medical Mission Board, Lima, Peru; 3 Catholic Medical Mission Board, New York, NY USA

**Keywords:** Women’s empowerment, Community health agents, Qualitative, Community health, Global health

## Abstract

Community health agents (CHAs) play a critical role in primary healthcare delivery and health promotion in low-resource settings. Though there is substantial evidence of the benefits of CHAs in achieving targeted community health outcomes, there is limited research into the impact of empowerment experienced by CHAs themselves. This study examined how working as a CHA impacts the lives and self-perceptions of women in Peru volunteering with Catholic Medical Mission Board’s (CMMB) markedly successful robust CHA model. We conducted six focus group discussions (FGDs) of 53 CHAs who implement CMMB programming in Trujillo and Huancayo, Peru. The FGDs were designed to explore themes related to empowerment, changes in women’s lives, and perceptions of themselves. We identified four major themes related to women’s empowerment: achievements, agency, meaningfulness, and resources. The most common empowerment theme was achievements, expressed through subthemes of changes in family behavior, self worth, education, health and nutrition, and rights and politics. The second most common empowerment theme was agency, with subthemes related to increases in using their voice, confidence, decision making, and participation. CHAs also reported experiencing empowerment through enhanced meaningfulness. CMMB’s CHA model is an example of how well-structured community programs can facilitate women’s empowerment. Providing meaningful community leadership opportunities can have far-reaching effects on women’s perceptions of themselves as valuable, capable, and empowered leaders. This work deepens our understanding of how to practically improve community health through empowering women to catalyze gender equality in communities with disproportionate barriers and limited opportunities burdening them.

## Background

The community health worker (CHW) model plays a critical role in primary health care delivery, particularly in low- and middle-income countries. This system of healthcare delivery is based on community empowerment, where natural leaders from within a community receive training and then spread health promotion messages, counseling, and basic healthcare [1]. The CHW model has been seen as a practical response to the challenging conditions of health provision in low-income settings [2] and also a way to strengthen health systems more generally [3]. The community health agents (CHAs) who implement CHW programming have been shown to be integral in building trust and rapport with patients, increasing health care access, and implementing countless health projects worldwide [4].

Community health agents (CHAs) are used around the world to improve community health and awareness. In the United States, CHAs have increased community participant knowledge on their diabetic disease processes [5]. In Sub-Saharan Africa, CHAs have been integral in extending and scaling up coverage of health services to help treat human immunodeficiency virus through antiretroviral therapy to improve health outcomes [6]. CHAs have also been involved in hypertension management through the motivation and empowerment of community members [7]. For some women, working as a CHA provides work experience and job skills that afford an opportunity to find paid work outside their homes. For example, in India, CHAs are paid a monthly salary by their government which increases career prospects, especially within governmental positions [8].

The majority of CHA research is focused on the impact CHAs have on their communities; there is less focus on the CHAs themselves and how their work impacts their perceptions of themselves. This project proposes to fill the gap in our understanding of the transformations that can happen to the CHA herself, as well as overall perceptions of women’s roles in society, by examining a project of the Catholic Medical Mission Board (CMMB), an organization that utilizes a robust CHA model. CMMB is an international non-governmental organization that provides medical and developmental aid to communities unduly affected by poverty and healthcare inequities. In 2012, a new project was undertaken by CMMB in two underserved areas of Peru, peri-urban settings of Huancayo and Trujillo. The project, entitled Taking Care of Your First 1000 Days in a Healthy Environment (hereinafter referred to as ‘First 1000 Days’) is a strategy to promote the health of mothers and their newborn children centered on the prevention and treatment of anemia and chronic malnutrition. Since the implementation of this project, anemia rates have fallen in areas where the First 1000 Days program is located by approximately 37%, substantially higher than the national recovery rate of 15% [9].

CHAs who implement the First 1,000 Days program become champions for change within their community, despite a number of social and domestic barriers: in parts of Peru, a common belief is that a woman’s place is in her home taking care of her spouse and children. Machismo culture in the form of male dominance over women, traditional gender roles, economic differences in pay between men and women, and intimate partner violence is highly pervasive as an example of gender inequality in Peru [10]. Society in general finds violence justified when women do not fulfill their roles, including not asking permission to go out or spending too much time outside the home. In 2015, 48.6% of women in Peru reported physical violence in their lifetimes,this statistic increased to 61% in rural areas of Peru [11].

The purpose of this study is to explore how working as a community health agent (CHA) in Peru has impacted women's lives and perceptions of themselves through qualitative data analysis. Understanding how women’s experiences modify their perceptions of themselves and other women in society can help to understand the process of shifting community norms and creating an environment that fosters women’s empowerment. There are many possible definitions of empowerment but for this study, we define empowerment as the increase of power and perception of a group that is currently at a power disadvantage.

## Methods

This project utilized a community-based participatory research approach in which both the community partners and researchers collaborated on all aspects of study conception, development, implementation, and analysis. We collected qualitative data to understand the experiences of CHAs working in Peru and how their work impacts their lives. We chose qualitative analysis of focus group discussions as this is an ideal method to understand and explore social norms, experiences, beliefs and opinions of a group or community [12].

### Participants and Data Collection

In March 2020, we conducted six 90-min focus group discussions (FGDs) in Huancayo and Trujillo, Peru with current and former community health agents (CHAs). There were a total of 53 Peruvian women participating in the FGDs, 21 in Trujillo and 32 in Huancayo. The study population included women who had worked as CHAs with CMMB in Huancayo and Trujillo, Peru for at least one year prior to the study. Both former and current First 1000 Days program CHAs were eligible to participate in the focus groups. The CMMB Peru research assistant invited CHAs to participate. After gaining consent, CHAs were divided into one of three groups based on the number of years they had worked as a CHA: 1–3 years, 3–5 years, and more than 5 years at each location (a total of six focus groups). Each CHA received a package of food staples as compensation for their participation; CMMB partners and support staff determined that this was the most appropriate form of compensation for participants. The number of participants in each focus group ranged from 6 to 11, with an average of 9 CHAs participating in each group.

The FGD guides were designed to explore how becoming a CHA has impacted their lives. Guided by the Most Significant Change Technique [13], the development of the FGD guide was aimed at finding themes related to personal empowerment and community perceptions and norms to explore the significance of how CHAs experiences impact the women themselves and the greater impact they may have on society to change social norms.

Discussions were conducted in Spanish and were audio-recorded, allowing data to be transcribed and translated into English. NaTakallam, a transcription/translation organization that employs refugees from around the world [14], transcribed the audio recordings and translated them for use in the data analysis process. The focus groups were conducted by a trained moderator who used the FGD guide to facilitate the discussion process, which lasted approximately 90 min each.

### Analysis

Two of the authors, EMA and MEK, performed a systematic and comparative analysis of the data as a basis for thematic analysis. Prior to the analysis, EMA and MEK engaged in epistemological reflexivity by considering any potential implications their existing biases and assumptions may have on the coding process [15], as well as by developing strategies for maintaining reliability and consistency in coding and analysis to address these potential implications. Both authors evaluated the 6 FDG transcripts independently to identify patterns and emerging themes before developing a codebook to represent initial areas of exploration. During this process, they incorporated Lee and Koh’s descriptions of specific dimensions of empowerment which address both cognitive and psychosocial factors impacting personal empowerment [16]. Once representative themes were identified and agreed upon, coders developed a coding system and separately coded the transcripts. Coding use and application were compared and discussed at each stage of the coding process to ensure consistency, as well as to facilitate the ongoing assessment of interrater reliability. If there were inconsistencies or disagreements in the application of codes, the codebook was clarified, refined, and updated, supporting consistent and accurate code use to increase credibility. Coders used NVIVO version 12, release 1.5 [17] to analyze transcript data, identify representative quotes relating to themes in the codebook, and interpret results.

## Results

All 53 participants spoke Spanish, with 14 of them speaking Quechua in addition to Spanish. The average number of years of CHA experience was 5.8 years for women in Trujillo and 4.5 years in Huancayo. Among participants in Trujillo, the highest level of education obtained was technical college (N = 6), but the majority of women listed secondary education as their highest level of education achieved (N = 10). Participants in Huancayo reported higher levels of education overall, listing University being the highest level of education accomplished (N = 5), with technical college being the level of education reported the most (N = 16). Additional characteristics of the study population can be found in Table 1.

**Table 1:** Demographic characteristics of study participants

	Trujillo N = 21	Huancayo N = 32
Age (Mean)	39.7	39.1
Years working as CHA (Mean)	5.8	4.5

	N (%)	N (%)
*Education*		
None	1 (5)	1 (3)
Primary	4 (19)	3 (9)
Secondary	10 (48)	7 (22)
Technical	6 (29)	16 (50)
Missing		5 (16)
*Marital status*		
Single	5 (29)	2 (6)
Cohabitating relationship	9 (43)	13 (42)
Married	7 (33)	16 (50)
Divorced	0	1 (3)
Prior work experience (Yes)	9 (43)	14 (44)

Demographic information including mean age, mean years working as a CHA, education level, marital status, and prior work experience of CHAs in Trujillo and Huancayo

Within the area of focus of women’s empowerment, four major themes emerged: (1) achievements, (2) agency, (3) meaningfulness, and (4) resources. The majority of the 409 coded themes were categorized as achievements (N = 215), followed by agency (N = 125), meaningfulness (N = 56), and resources (N = 13). Each of the themes and subthemes are described in detail below. A summary of our findings can be found in Table 2.

**Table 2:** Major themes and subthemes identified in focus group discussions

Personal empowerment (N = 409)^a^			
Theme	N^b^	Subtheme	N^b^
Achievements	215	Family Behavior	75
		Self Worth	49
		Education	44
		Health & Nutrition	33
		Rights & Politics	14
Agency	125	Voice	62
		Confidence	31
		Decision Making	20
		Participation	12
Meaningfulness	56		
Resources	13	Social	12
		Human	1
		Material	0

^a^There were a total of 409 themes related to personal empowerment identified in the coded transcripts

^b^N indicates number of times each theme and subtheme were coded in all six focus group discussions

### Achievements

The sub themes that emerged describing achievements were: (1) family behavior, (2) self worth, (3) education, (4) health and nutrition, and (5) rights and politics. The most common sub theme was family behavior (N = 75), pursued second by self-worth (N = 49), followed by education (N = 44), then health and nutrition (N = 33), with rights and politics last (N = 14).

One common description that came up with *family behavior* was reflections on family dynamics. Family behavior encompasses the social norms that direct how families interact with one another. This is highly influenced by the Peruvian machismo culture. Family behavior was sometimes used in reference to increasing support from husbands and prioritizing family values.

Little by little he started to help me, now he picks up a broom and sweeps. At first, he didn't want his son to do the bed, he said that his son is a man... I talked to him about the things [CHAs] learned, and now he sends his son to help me. He is changing. (Trujillo)

*Self-worth* was coded to CHAs observations of internal changes and perceptions of increased recognition of their worth and value. Several women remarked improvements in their self-esteem as a result of their involvement with CMMB. Several experiences described the impact of their community role and connections with others on their self worth.

I feel fulfilled, I feel like a leader in my community…I see that I can support [them and] my family, and help them with anything and any doubts they may have about health. (Trujillo)

*Education* was referenced by several CHAs in reports of horizontal learning that had occurred between other CHAs and community members. Education here encompasses learning applicable skills and gaining relevant knowledge. Education was also sometimes coded in reference to internal changes and the resulting impact that CHAs described as coming with knowledge.

I have learned a lot through the workshops...there [have] been a lot of ideas exchanges [between CHAs], and I practice it with my family and [with the] mothers. (Huancayo)

Instances coded to *health and nutrition* were sometimes characterized by themes of learning and gaining awareness of foods and actions that can result in an increase in health. Several CHAs expressed changes they had made to improve health and nutrition behaviors as a result of this learning.

Now that [I see] the psychologist...I have learned about [healthy family communication], so I go home and say...we need to change. And as a community healthcare worker, I need to change first myself so I can guide my neighbors, right? (Huancayo)

*Rights and politics* was used to code descriptions of positive outcomes gained through advocacy or perceived entitlement to equitable treatment.

Now I feel that I have strength, courage, I value myself, men and women have the same rights. And I support my family even more strongly. (Trujillo)

### Agency

The sub themes that emerged related to agency were: (1) voice, (2) confidence, (3) decision making, and (4) participation. Of these subthemes, voice (N = 62) was the most commonly referenced idea, followed by confidence (N = 30), then decision making (N = 20) and finally, participation (N = 12).

*Voice* was coded to instances of advocacy or actions that influenced agency and positive outcomes. Voice is defined as the ability to express opinions or attitudes openly. A common narrative that emerged with the CHAs was using their voice to advocate for rights within the community. Several CHAs shared their experiences of advocating for themselves and their peers in healthcare settings.

I felt powerless, but...when I joined the CMBB, they gave us the training regarding health care rights...I went to back to the center [where they mistreated my son], and I said: “You know what, bring me the complaint book because I will write mine down.” ...I said to them, …”I believe that [because] he is a human being, you need to [provide treatment]....how is it possible that you are treating us like this?” (Huancayo)

*Confidence* was coded to instances in which CHAs expressed having belief in their abilities. Confidence encompasses self-assurance and belief in one’s self and abilities. Some CHAs shared the positive impact that being confident in their information and knowledge had on their community interactions.

I didn't have much trust [from] the community, but now that I am a community agent I do have more trust with the mothers...I also tell them what information I receive...I share with them with more confidence. (Trujillo)

*Decision making* was coded when participants described their process in making strategic life choices or factors that influenced their decisions. Many women reported gaining the ability to make choices about themselves and their family. Some participants gave examples of how the CHA training led to them gaining knowledge that facilitated their ability to make choices related to advocacy and health.

I feel confident in making decisions. Because [of] the trainings...I know [which] symptoms [need us to visit] a health center...I am responsible for my children. (Trujillo)

It was also coded to larger strategic actions that some CHAs had taken to improve their lives, such as one instance in which a CHA described her decision to leave her abusive husband.

I gave myself the opportunity to say no...it ends here. And since [he was holding me back]...I was strong and said NO… [CMMB] never abandoned me. (Huancayo)

*Participation* was coded to descriptions of community involvement that resulted in achievements, positive outcomes, or increased agency. Multiple CHAs reported an increase in taking part in their communities. Several CHAs shared ways in which they became involved with their communities by providing others with support.

I’m more involved with the community around me. Seeing how they rely on me...and being able to respond, makes me feel like there are new doors opening since I’m now able to help and work with them better than ever before. (Huancayo)

### Meaningfulness

Meaningfulness was coded to descriptions of aspects of working as a CHA that aligned with their values, felt internally rewarding, or increased agency. For many CHAs, working with CMMB and providing services to their community gave them significance in their lives. Common examples given were descriptions of the connections they build within the community, and in recognizing the value of their support and knowledge.

Being able to see the change in the health of the people and that the mothers are applying what we teach them...that’s my motivation, seeing that you are moving forward step by step. (Huancayo)

### Resources

The theme of resources was coded to descriptions of preconditions that enhanced CHAs ability to exercise choice. Within the theme of resources, several sub themes emerged: (1) social, (2) human, and (3) material. There was a clear divide in the coding frequency between social (N = 12), human (N = 1) and material (N = 0) resources.

The items that were coded to the sub theme of *social resources* were resources that women had gained access to as a direct result of connections they had made through being a CHA, as well as their perceptions of changes in opportunities and agency as a result of this. Another common experience was related to community building through CHA work. Some participants discussed how changes in their relationships with other community members and connections they have built with the mothers that they visit are a result of mutually sharing information and spending more time out in the community.

I’m more involved with the community...there are new doors opening...not only because of my training but also because of the connections I’ve made (Huancayo)

## Discussion

This qualitative study identified that women found significant thematic commonalities in personal empowerment after working as a CHA in their communities for over a year. This has important implications about their perceptions within themselves and their family. Of the women interviewed, there were various reports of substantial indirect changes in their personal lives after engaging with CMMB’s program in terms of their own self-realization as a person of worth in this machismo social order.

We identified that women recognized substantial increases in achievement in terms of family behavior, feelings of self worth, and education after working as a CHA. In rural areas in particular, there has been slow progress for women’s achievement, a vital root in feminism; for achievement to work towards confronting the patriarchy, achievement must lead to financial and economic independence, democratic inclusion, education, future aspirations, increases in decision-making, public interactions, community inclusion, and internal shifts in perception [18–22]. Our work emphasizes that engagement with the community results in visible increases in personal empowerment, an improvement towards gender equality despite minimal direct benefits (e.g. wages, voting, etc.). We found that when women were provided the opportunity to work as a CHA, they were also provided a path to other achievements including gaining status within their family and becoming leaders in their community. This has a substantial impact on the women’s personal and family values. Therefore, if women are given an opportunity to work as a CHA, it will have a multitude of indirect effects towards women’s empowerment.

Women working as CHAs report an increase in personal agency in using their voice to advocate for themselves. Women felt more confident in their decision making and as a result participated in more community decision making. Agency through women’s voices is a step towards progress for women; education, formal representation in positions of power, recognition of women’s voices in the legal sector are all needed to protect women from being taken advantage of by systemic oppression in the form of gender inequalities [23–25]. Though other studies are focused on paid and formal positions that increase the role of agency [18, 22], our study shows that volunteering as a CHA provides the opportunity for women’s voices to be heard. Personal agency gives direction and urgency to women to make decisions in their personal lives from how they spend their time to how they interact with the community, thus narrowing the gap in gender inequality [23, 26].

Meaningfulness is the third most coded phrase we found in our FGDs. Self-knowledge and development are key reasons why unpaid volunteer work is central to volunteers’ lives and their perceptions of themselves, their identities, and overall life satisfaction; social commitment, endorsing one’s character strengths, and altruism for one’s community are present in many volunteers who dedicate their time to others [27, 28]. The CHAs in this program exhibit these traits as evidenced by their statements and quotes during the FGDs, they are committed to their communities and give their time and knowledge to develop themselves and other women to find overall meaning with their work.

In terms of resources, the CHAs did not report any material benefits including financial gain, goods, or other physical resources; however, they did report gains in social resources. Other CHA research demonstrates that material gain seems to be a necessity for women’s empowerment and CHA program success by mitigating attrition rates and fostering community relations [29]. Our work partly contradicts this supposition as the material benefits do not seem to be a major factor in this community in CHA retention and program effectiveness, results from this study show a model where material and other traditional forms of gain are not necessary to obtain personal empowerment and program success. Well structured community programs can foster program success and women’s empowerment.

The CHAs in this program are experiencing remarkable benefits from their role in their communities particularly in terms of their own perceptions of themselves. For CHAs, there is a clear sense of pride in their work. More importantly, perhaps, they feel a sense of empowerment and can see, sometimes for the first time, that they are valuable members of society and capable of accomplishing extraordinary things. This feeling of empowerment can have major societal implications: not only do empowered women fight for equality, they can be a model for adolescent girls to show that an alternative future is possible in parts of the world where there are limited opportunities for young women and girls. In Peru, especially in rural areas, poverty, adversity and household roles are particularly prevalent among the adolescent population; for many children, especially females, the path of working in the household begins when they are young and continues into adulthood [30]. For them, working as a CHA represents an alternative way of life to exit this patriarchal system. Considering Peru is a middle-high income country with deep inequalities, analyzing CMMB Peru’s model provides important lessons on how to address public health challenges of those left behind within emerging economies.

A number of limitations need to be considered for this study. First, we do not have a baseline of women’s perceptions of empowerment before working as CHA. Thus, we cannot measure a change in levels of empowerment before and after CHA work. However, our FGD guide was structured such that we focused on women’s perceptions on how working as a CHA with CMMB directly impacted their lives. The discussion focused on how women felt they had changed since working with CMMB. We also recognize potential selection bias, as women had at least one year of CHA experience to participate in the study. Those who do not remain in the CHA program may not feel this sense of change; however, a majority of CHAs do stay longer than a year in the CMMB program. Therefore, we do not believe any level of selection bias is affecting our reported results. This study was conducted in Peru, a middle-high income country and considered a machista society, meaning the generalizability of this study may be limited. Further, CMMB has a particularly robust CHA model compared to various models of CHWs throughout the world and thus other CHA models may not find the same level of empowerment when replicating this work.

Working as a community health agent (CHA) can empower women with ultimate benefits to the lives of community women in low resource settings. The indirect benefits of being a CHA has been shown to drastically improve the perceptions women have of themselves and therefore, lead to powerful examples of leadership and empowerment for themselves and other women*.* Moreover, the project expands on current literature to examine how empowering women to act as CHAs can ultimately improve the lives of women in low resource settings. The CHW model not only improves health in communities but can have additional far reaching impacts on women’s lives as it has the ability to influence perceptions and empower women.

## Data Availability

The datasets generated and analyzed during the current study are not publicly available due to confidentiality and privacy for participants but data collection tools available from the corresponding author on reasonable request.

## References

[1] Cherrington A, Ayala GX, Elder JP, Arredondo EM, Fouad M, Scarinci I (2010). Recognizing the diverse roles of community health workers in the elimination of health disparities: From paid staff to volunteers. Ethnicity & Disease.

[2] Adam MB, Dillmann M, Chen M, Mbugua S, Ndung’u J, Mumbi P, Waweru E, Meissner P (2014). Improving maternal and newborn health: Effectiveness of a community health worker program in rural Kenya. PLoS ONE.

[3] Kamuzora P, Maluka S, Ndawi B, Byskov J, Hurtig A-K (2013). Promoting community participation in priority setting in district health systems: Experiences from Mbarali district, Tanzania. Global Health Action.

[4] Rosenthal EL, Brownstein JN, Rush CH, Hirsch GR, Willaert AM, Scott JR, Holderby LR, Fox DJ (2010). Community health workers: Part of the solution. Health Affairs.

[5] Norris SL, Chowdhury FM, Van Le K, Horsley T, Brownstein JN, Zhang X, Jack L, Satterfield DW (2006). Effectiveness of community health workers in the care of persons with diabetes. Diabetic Medicine.

[6] Hermann K, Van Damme W, Pariyo GW, Schouten E, Assefa Y, Cirera A, Massavon W (2009). Community health workers for ART in sub-Saharan Africa: Learning from experience—Capitalizing on new opportunities. Human Resources for Health.

[7] Brownstein JN, Chowdhury FM, Norris SL, Horsley T, Jack L, Zhang X, Satterfield D (2007). Effectiveness of community health workers in the care of people with hypertension. American Journal of Preventive Medicine.

[8] Prasad, B. M., & Muraleedharan, V. (2007). Community health workers: A review of concepts, practice and policy concerns. A review as part of ongoing research of International Consortium for Research on Equitable Health Systems (CREHS).

[9] Catholic Medical Mission Board. Improving access to better nutrition 2019. Retrieved April 30, 2019, from https://cmmb.org/programs/projects-3/nutrition/

[10] Davila KA (2019). The impact of machismo on women’s health and security in Peru and how the state fails women.

[11] Benavides M, Leon Jara Almonte J, Ponce de Leon Marquina M (2015). The co-occurrence of domestic and child violence in urban Peru: Evidence from three regions. Journal of Family Violence.

[12] Wong LP (2008). Focus group discussion: A tool for health and medical research. Singapore Medical Journal.

[13] Davies R, Dart J (2007). The “most significant change” (MSC) technique: A guide to its use.

[14] *Translation Services*. (n.d.). Natakallam.com; NaTakallam. Retrieved December 1, 2021, from https://natakallam.com/translation-services/

[15] Dowling M (2006). Approaches to reflexivity in qualitative research. Nurse Researcher.

[16] Lee M, Koh J (2001). Is empowerment really a new concept?. The International Journal of Human Resource Management.

[17] QSR International Pty Ltd. (2018). NVivo (Version 12, Release 1.5). https://www.qsrinternational.com/nvivo-qualitative-data-analysis-software/home

[18] Mandal, K. C. (2013). Concept and types of women empowerment. *A Formerly Scholar of Vidyasagar University, Department of Political Science with Rural Administration, Midnapore, West Bengal, India*, *9*(2), 17–30. http://americanscholarspress.us/journals

[19] Nayak P, Mahanta B (2008). Women empowerment in India. SSRN Electronic Journal.

[20] Sundaram MS, Sekar M, Subburaj A (2014). Women empowerment: Role of education. International Journal in Management and Social Science.

[21] Tandon T (2016). Women empowerment: Perspectives and views. International Journal of Indian Psychology.

[22] Varghese T (2011). Women empowerment in Oman: A study based on women empowerment index. Far East Journal of Psychology and Business.

[23] Briones, L. (2009). *Capability and international labor migration for domestic work. *In *Empowering migrant women: Why agency and rights are not enough*. Essay, Routledge.

[24] Kane BC, Williamson F (2016). Women, agency and the law, 1300–1700.

[25] Klugman J, Hanmer L, Twigg S, Hasan T, McCleary-Sills J, Santamaria J (2014). Voice and agency: Empowering women and girls for shared prosperity.

[26] United Nations. (n.d.). *Achieve gender equality and empower all women and girls*. United Nations. Retrieved March 9, 2022, from https://sdgs.un.org/goals/goal5

[27] Littman-Ovadia H, Steger M (2010). Character strengths and well-being among volunteers and employees: Toward an integrative model. The Journal of Positive Psychology.

[28] Schnell T, Hoof M (2012). Meaningful commitment: Finding meaning in volunteer work. Journal of Beliefs & Values.

[29] de Vries DH, Pool R (2017). The influence of community health resources on effectiveness and sustainability of community and lay health worker programs in lower-income countries: A systematic review. PLoS ONE.

[30] Crivello G, Boyden J (2012). On childhood and risk: An exploration of children's everyday experiences in rural Peru. Children & Society.

